# Two SNP Mutations Turned off Seed Shattering in Rice

**DOI:** 10.3390/plants8110475

**Published:** 2019-11-06

**Authors:** Yu Zhang, Jiawu Zhou, Ying Yang, Walid Hassan Elgamal, Peng Xu, Jing Li, Yasser Z. El-Refaee, Suding Hao, Dayun Tao

**Affiliations:** Yunnan Key Laboratory for Rice Genetic Improvement, Food Crops Research Institute, Yunnan Academy of Agricultural Sciences (YAAS), Kunming 650200, China; zhangyu_rice@163.com (Y.Z.); zhjiawu@aliyun.com (J.Z.); yaasyang@126.com (Y.Y.); elgamal.rrtc@gmail.com (W.H.E.); xupeng@xtbg.ac.cn (P.X.); lijinglab@163.com (J.L.); elrefaeey@yahoo.co.in (Y.Z.E.-R.); haosuding@126.com (S.H.)

**Keywords:** Seed shattering, *O. barthii*, *O. sativa*, *HS1*, haplotype

## Abstract

Seed shattering is an important agronomic trait in rice domestication. In this study, using a near-isogenic line (NIL-*hs1*) from *Oryza barthii*, we found a hybrid seed shattering phenomenon between the NIL-*hs1* and its recurrent parent, a *japonica* variety Yundao 1. The heterozygotes at *hybrid shattering 1* (*HS1*) exhibited the shattering phenotype, whereas the homozygotes from both parents conferred the non-shattering. The causal *HS1* gene for hybrid shattering was located in the region between SSR marker RM17604 and RM8220 on chromosome 4. Sequence verification indicated that *HS1* was identical to *SH4*, and *HS1* controlled the hybrid shattering due to harboring the ancestral haplotype, the G allele at G237T site and C allele at C760T site from each parent. Comparative analysis at *SH4* showed that all the accessions containing ancestral haplotype, including 78 wild relatives of rice and 8 African cultivated rice, had the shattering phenotype, whereas all the accessions with either of the homozygous domestic haplotypes at one of the two sites, including 17 wild relatives of rice, 111 African cultivated rice and 65 Asian cultivated rice, showed the non-shattering phenotype. Dominant complementation of the G allele at G237T site and the C allele at C760T site in *HS1* led to a hybrid shattering phenotype. These results help to shed light on the nature of seed shattering in rice during domestication and improve the moderate shattering varieties adapted to mechanized harvest.

## 1. Introduction

During rice domestication, seed shattering is one of the most greatly changed traits for seed dispersal. Easy shattering leads to the loss of production [1], and more attention is paid on selection for non-shattering but threshable rice in modern rice breeding [2]. Seed shattering is caused by the formation and degradation of the abscission zone (AZ), which, constituted by a band of small cells, is responsive to signals promoting abscission [3].

Recently, several genes responsible for seed shattering were identified in rice. *qSH1* encodes a BELL homeobox protein. An SNP mutation in the regulatory region of *qSH1* could inhibit its expression, which resulted in defective abscission layer development [4]. The allelic genes of *SH4* [5], *SHA1* [6] and *GL4* [7] encoding a trihelix transcriptional factor, all controlled the seed shattering, however, haplotypes were divergent because of two different SNP mutations. A “G237T” mutation in *SH4* and *SHA1* was responsible for the loss of seed shattering in Asian cultivated rice [5,6], whereas “C760T” transition in *GL4* conferred non-shattering seeds in African cultivated rice [7]. The *SHAT1* gene, which encoded an APETALA2 transcription factor, was responsible for seed shattering through specifying abscission zone development in rice. The expression of *SHAT1* was positively regulated by the transcription factor *SH4,* which was required for the AZ identification during the early spikelet developmental stage, and *qSH1* functions downstream of *SHAT1* and *SH4*, promoting the AZ differentiation by maintaining the expression of *SHAT1* and *SH4* [8]. *SH5*, which is highly homologous to *qSH1*, also controlled seed shattering by regulating lignin deposition in the pedicel region [9]. *OsGRF4* could increase the expression of two cytokinin dehydrogenase precursor genes resulting in the high cytokinin level, which led to reduced seed shattering [10]. *OSH15* together with *SH5* induced seed shattering by repressing lignin biosynthesis genes [11]; *ObSH3* in *Oryza barthii* encoded a YABBY transcription factor, which was also required for the development of the seed abscission layer [12]. In addition, some other minor genes and allelic interaction at major locus might be involved in the seed shattering domestication as rice underwent a prolonged domestication process, with continuing selection for reduced shattering [13,14].

*O. barthii* is one of the relatives distributed in West Africa, sharing the same AA genome as Asian cultivated rice. Most of *O. barthii* accessions exhibited the seed shattering. The previous report indicated that *GL4* in *O. barthii* was involved in the non-shattering selection during the African cultivated rice domestication [7], but the nature of seed shattering was still not clear. Here, we report that a novel locus, named *hybrid shattering 1* (*HS1*)*,* controlled the seed shattering in the hybrid between *O. barthii* and *O. sativa*. A near-isogenic line (NIL-*hs1*) from *O. barthii,* and its recurrent parent, a *japonica* variety Yundao 1, showed the non-shattering phenotype. Interestingly, the hybrid between two parents showed the seed shattering, similar to the ancestral wild rice. Whether it was shattering or not was dependent on the different haplotypes of two SNPs at *HS1*. This result could help us understand the complex molecular mechanism of seed shattering.

## 2. Results

### 2.1. HS1 Controlled the Hybrid Seed Shattering in Rice

**Figure 1:**
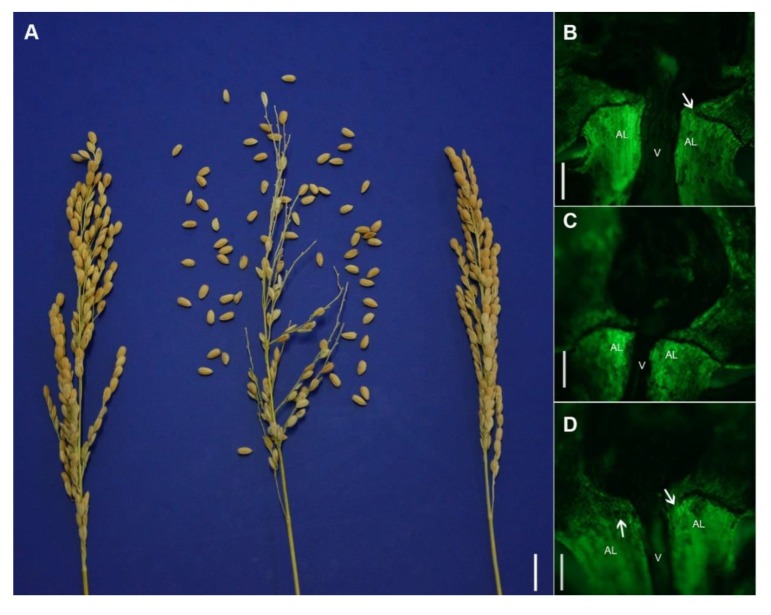
(**A**) The seed shattering rate of Yundao 1 (left), F_1_ hybrid (middle) and near-isogenic line (NIL-*hs1*) (right). Scale bars = 0.5 cm. (**B–D**) Fluorescence images of a longitudinal section of the spikelet and pedicel junction in Yundao 1, F_1_ hybrid and NIL-*hs1*, respectively. (**B**) Yundao 1 showed an incomplete in abscission zone. (**C**) F_1_ hybrid with a complete abscission layer. (**D**) NIL-*hs1* exhibited a deficiency in abscission layer on the palea side and partial abscission layer on the lemma side. AL: Abscission layer, V: Vascular bundle. White arrow indicates a deficiency in abscission zone. Scale bars = 10 µm.

We developed a NIL-*hs1* carrying genome fragment from *O. barthii* on chromosome 4 in the Yundao 1 genetic background. Surprisingly, Yundao 1 and the NIL-*hs1* showed the non-shattering seed, while F_1_ hybrid exhibited seed shattering (Figure 1A, Table S1). In order to distinguish the differences in abscission layer structure between F_1_ hybrid and its parents, longitudinal sections at the seeds base were observed using fluorescent microscopy. The results showed that Yundao 1 displayed the deficiency in abscission zone development near the vascular bundle (Figure 1B), the NIL-*hs1* showed no abscission layer on the palea side and the partial abscission layer on the lemma side between the seed pedicel and the spikelet, respectively (Figure 1D). Conversely, the F_1_ hybrid had a continuous abscission zone between the vascular bundle and the epidermis (Figure 1C). These results indicated that seed shattering in the F_1_ hybrid resulted from the complete and continuous abscission layer, whereas the loss of seed shattering in Yundao 1 and NIL-*hs1* was caused by the irregular development of the abscission zone.

### 2.2. Dominant Complementation of G Allele at G237T Site and C Allele at C760T Site in HS1 Led to Hybrid Seed Shattering Phenotype

In order to understand whether *HS1* acted as a single Mendelian factor or not, the seed shattering rate was investigated in BC_4_F_1_ and BC_4_F_2_ populations derived from the cross between Yundao 1 and NIL-*hs1*. All the BC_4_F_1_ individuals showed seed shattering. The seed shattering rate in the BC_4_F_2_ population was segregated into non-shattering and shattering classes in a 246:205 ratio, which fitted the 1:1 ratio (χ^2^ = 1.870, *P* = 0.172) (Figure S1). These results indicated that the seed shattering in F_1_ hybrid was controlled by a single gene. We designated it as *HS1*.

**Figure 2:**
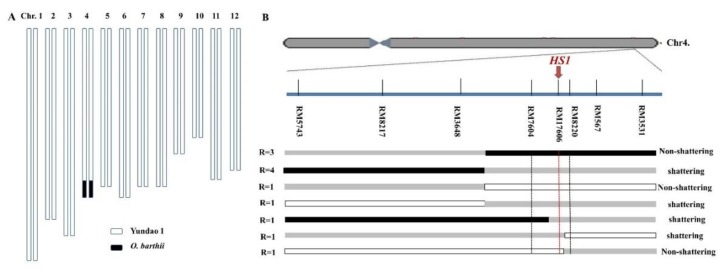
(**A**) Graphical genotypes show that an *O. barthii* chromosomal segment was introgressed into the NIL-*hs1* genome on chromosome 4. (**B**) Genetic mapping of *hybrid shattering 1* (*HS1*) on Chromosome 4, white bar: homozygous Yundao 1; grey bar: heterozygous; black bar: homozygous NIL-*hs1*. “R” means the number of recombinants.

**Figure 3:**
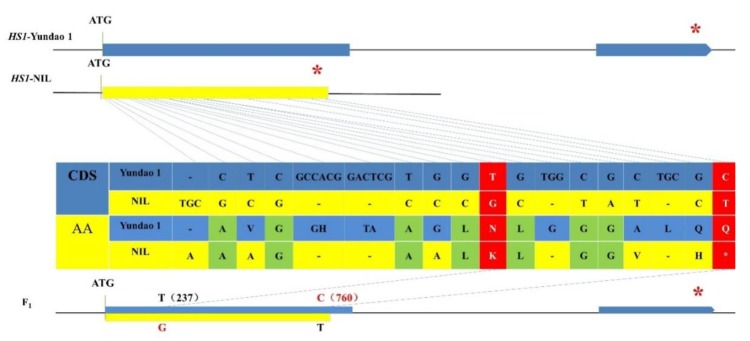
The difference in the coding sequence and amino acid of *HS1* between Yundao 1 and NIL-*hs1*. Synonymous mutations and functional mutations were shown in green and red, respectively. The asterisk indicates the stop codon.

A population of 790 BC_4_F_2_ plants was generated for mapping the *HS1.* Eight polymorphic SSR markers in the introgressed region on chromosome 4 were used for genotyping the 790 individuals in the BC_4_F_2_ population. *HS1* was mapped into a 0.4 cM region flanked by RM17604 and RM8220, at genetic distances of 0.3 and 0.1 cM, respectively, and co-segregated with RM17616 (Figure 2). The homozygotes from both parents at RM17616 showed a non-shattering phenotype, whereas the heterozygotes at RM17616 showed a shattering phenotype. Based on the GRAMENE public database (http://www.gramene.org), the physical distance between RM17604 and RM8220 was about 434.6 kb. The mapping region of *HS1* was similar to the location of *SH4/GL4* (LOC_Os04g57530/ORGLA04G0254300) identified from the Asian rice and the African rice [4,5,6]. In order to confirm whether the *HS1* was allelic to *SH4/GL4* or not, the sequence analysis of *SH4* in Yundao 1 and NIL-*hs1* was performed. A total of 13 SNPs, 5 indels in the 2.3 kb of aligned sequenced DNA were identified (Figure 3), which resulted in 1 amino acid insertion, 4 amino acid substitutions, 6 amino acid deletions and pre-stop codon in NIL-*hs1*, respectively (Figure 3). Of these, two base substitutions of G237T and C760T (C760T referred that the C to T SNP mutation in *HS1* was at nucleotide position 760 in *O. barthii*, which was the same as C769T mutation in Yundao 1) resulted in the mutation of Asn79 to Lys79 and Gln258 to a stop codon, respectively. It was reported that the G allele at G237T site and the C allele at C760T site were responsible for the seed shattering during the Asian cultivated rice and the African cultivated rice domestication, respectively [4,5,6]. Thus, we postulated that *HS1* was identical to *SH4* and *GL4*. The G allele at G237T site and the T allele at C760T in the Yundao 1 background, and the T allele at G237T site and the C allele at C760T in the NIL-*hs1* background all conferred the non-shattering phenotype, whereas the combination of the G allele at G237T site and the C allele at C760T site exhibited the shattering phenotype. Dominant complementation of the G allele at G237T site and the C allele at C760T site in *SH4* led to the hybrid shattering phenotype. Moreover, *SH4* in NIL-*hs1* had a unique deletion of the 227th amino acid residue isoleucine (Ile), compared with that in other AA genome species in the genus *Oryza*.

### 2.3. Two SNP Mutations Turned off Seed Shattering in Rice

In order to confirm the function of the two SNPs, we reanalyzed the gene sequence of *SH4* in 95 wild accessions of rice, 119 *O. glaberrima* and 65 *O. sativa* using previously published data. All the accessions harboring both the G allele at G237T site and the C allele at C760T site exhibited the shattering phenotype, including 2 *O. longistaminata* accessions, 22 of 28 *O. barthii* accessions, 8 of 119 *O. glaberrima* accessions, 2 *O. glumaepatula* accessions, 20 of 25 *O. nivara* accessions and 30 of 36 *O. rufipogon* accessions. All the accessions (varieties) with either the G allele at G237T site or the C allele at C760T site showed the non-shattering, including 6 of 28 *O. barthii* accessions, 111 of 119 *O. glaberrima* accessions, 5 of 25 *O. nivara* accessions, 6 of 36 *O. rufipogon* accessions and 65 Asian cultivated varieties. These results indicated that the G allele at G237T site and the C allele at C760T site were ancestral alleles in African rice domestication and Asian rice domestication, respectively, whereas the T allele at both sites that resulted from selection pressure were mutation alleles. The ancestral haplotype (the G allele at G237T site and the C allele at C760T site) induced a shattering phenotype in rice, whereas domestic haplotypes (the G allele at G237T site and the T allele at C760T site, the T allele at G237T site and the C allele at C760T site) all exhibited the loss or reduction of seed shattering (Table 1), which was consistent with our experimental results that the hybrid harboring ancestral haplotype showed a shattering phenotype; however, Yundao 1 and NIL-*hs1* carrying homozygous domestic haplotypes exhibited a non-shattering phenotype.

## 3. Discussion

What causes the differences in seed shattering in different species is totally an open question. Loss or reduction of seed shattering represents a key transition to domestication in rice [15,16]. In this study, it was a serendipitous finding that the hybrid F_1_ derived from the cross between the non-shattering Yundao 1 and NIL-*hs1* that displayed the seed shattering phenotype. And we reported that a novel locus *HS1* controlled the hybrid shattering between *O. sativa* and *O. barthii*. Yundao 1 and NIL-*hs1* showed the irregular and partially developed abscission layer, whereas the F_1_ hybrid exhibited a continuous abscission layer between seed pedicel and spikelet. *HS1* and *SH4* were mapped into a similar region on chromosome 4 [5], interestingly, *SH4* functioned in the seed shattering on the homozygous background, whereas *HS1* conferred the seed shattering on the heterozygous background. What resulted in this difference? It was suggested that an allelic interaction at a single locus or an epistatic interaction at two independent loci from the non-shattering species *O. sativa* and *O. barthii* determined the phenotype. Interestingly, *HS1* and *SH4* were mapped into a similar region on Chromosome 4 and sequencing analysis confirmed that *HS1*, allelic to *SH4*, carrying ancestral haplotype (the G allele at G237T site and the C allele at C760T site) contributed to shattering in the F_1_ hybrid. Thus, an allelic interaction at *SH4* was responsible for the hybrid seed shattering. NIL-*hs1* and most of *O. glaberrima* accessions shared the same *SH4* haplotype, and we also found that the hybrid between the *O. glaberrima* and *O. sativa* also displayed the seed shattering phenotype. As we know, it is difficult to obtain the hybrid progeny between *O. glaberrima* and *O. sativa*, one of the reasons is that interspecific hybrid sterility between *O. glaberrima* and *O. sativa* prevents the formation of hybrid offspring, and another reason is that strong seed shattering in the hybrid increases the difficulty of the crossing. Previous studies reported that one single-nucleotide polymorphism, G237T or C760T, controlled seed shattering in rice independently, because one SNP was fixed and not polymorphic in the wild and cultivated accessions [5,6,7]. In this study, the relationship of the different haplotypes of *SH4* and the shattering phenotype was analyzed from the whole gene sequence viewpoint. Moreover, two base mutations of G237T and C760T at *SH4* occurred in Trihelix DNA binding domain, indicating that this domain played an important role in seed shattering and either of the nucleotide acid mutations had no effect on the function of the DNA binding domain. The transcription factor *SH4* controlled the AZ identification by positively regulating the expression of *SHAT1,* and *qSH1* could promote the AZ differentiation by maintaining the expression of *SHAT1* and *SH4* [8], suggesting that two amino acid substitutions (Asn79 to Lys79 and Gln258 to a stop codon) in *SH4* might affect the interaction with *SHAT1* and *qSH1*, resulting in the loss of seed shattering. In addition, with the similar genetic background, the shattering degree of Yundao 1 was easier than that of NIL-*hs1*, there were two possibilities: (1) The haplotype of T237 and C760 combination conferred the easier shattering than that of G237 and T760 combination; (2) other genes in the introgressed region could decrease the seed shattering rate by regulating the expression of *SH4.* These results would provide new clues into the molecular basis of seed shattering in rice, and breeders can take the advantage of different haplotype combinations adapted to the moderate shattering degree so as to meet the need for mechanized harvest.

Asian cultivated rice (*O. sativa* L.) was domesticated from wild species *O. rufipogon* thousands of years ago [17,18], whereas *O. glaberrima* Steud. was an African species of rice that was domesticated from the wild progenitor *O. barthii* about 3000 years ago [15,16]. In this study, 79% of *O. barthii* accessions harbored the G allele at G237T site and the C allele at C760T site in *SH4*, but 93% of *O. glaberrima* accessions carried the G allele at G237T site and the T allele at C760T site. It is suggested that the wild-type G at the G237T site was fixed, while the mutated T allele at the C760T site was selected during the African cultivated domestication. The function of the G allele at G237T site (Lysine residue) may be critical for the growth and development of the African wild relative of rice and African cultivated rice so that it was fixed during the gradual domestication process, while the T allele at G237T site contributing to the small grain size was selected, which may be an adaptation to the extreme environment in West Africa, such as drought, soil acidity, iron and aluminum toxicity [7,19,20]. Two haplotypes of *SH4* in *O. rufipogon* existed, whereas the cultivated rice only had one domestic haplotype, these results were also observed in *O. barthii* and *O. glaberrima*. Compared with African cultivated rice, domestic allele in Asian cultivated rice was the G237T mutation, while the C allele at C760T mutation was fixed, indicating that the C alleles at the C760T site might be involved in the selection of a large grain size [7]. This might be one of the reasons that the yield of Asian cultivated rice was higher than that of African cultivated rice. Taken together, the seed shattering characteristics were selected in African cultivated rice and Asian cultivated rice, respectively, which were consistent with the theory that *O. glaberrima* and *O. sativa* were domesticated in parallel [12,18].

## 4. Materials and methods

### 4.1. Plant Materials

An *O. barthii* accession, Acc.104284, introduced from the International Rice Research Institute (IRRI), as a donor and male parent, was crossed with a temperate *japonica* variety of *O. sativa*, Yundao 1, from Yunnan province, P. R. China. The male gametes in hybrid F_1_ between *O. barthii* and *O. sativa* were fully sterile, and the female gametes in hybrid F_1_ were partially fertile, thus, hybrid F_1_ as the female parent was consecutively backcrossed with Yundao 1 as the male parent. BC_3_ plants were self-pollinated for 13 generations to produce the BC_3_F_14_ introgression lines. Four hundred and twenty-six polymorphic SSR markers evenly distributed on 12 chromosomes of rice were used to evaluate the substituted fragments from *O. barthii.* The results indicated that only 14.8 cM segments on chromosome 4 were substituted by the *O. barthii* genome. The individuals harboring the homozygous genome fragment from *O. barthii* were selected as NIL-*hs1*. NIL-*hs1* was crossed with the recurrent parent Yundao 1, and then was self-fertilized to produce the BC_4_F_2_ population. We found the BC_4_F_1_ plants all showed seed shattering, while seed shattering and non-shattering were both observed in the BC_4_F_2_ population. Seven hundred and ninety individuals were used to mapping *HS1* for seed shattering between *O. sativa* and *O. barthii.*

All plant materials were grown in the paddy field at the Experiment Station, YAAS, located in Jinghong, Yunnan Province, P. R. China.

### 4.2. Evaluation of Seed Shattering Rate

The spikelets of each plant were bagged at the stage of heading. Then, the panicles were collected at the stage of maturity, and panicles freely fell 2 m to the plastic box (60 cm × 90 cm). All the grains shredded prior to the test were counted as shattering grains. The shattering rate was calculated by a percentage of shattered seeds to the total seeds. The shattering rate below 5% or above 50%, was defined as the non-shattering type and the shattering type, respectively.

### 4.3. Microscopy

Seeds including pedicels were collected at the stage of maturity, and slices made by mature and dry seeds were stained with 1% acridine orange. Abscission layer at seed base was observed by Fluorescent microscopy (OLYMPUS BX53).

### 4.4. DNA Extraction and SSR Analysis

The experimental procedure for DNA extraction was performed as previously described [21]; rice SSR markers were selected from the Gramene database (http://www.gramene.org) or previously published SSR markers in rice [22]. PCR was performed as follows: a total volume of 10 μL containing 10 ng template DNA, 1 × buffer, 0.2 μM of each primer, 50 μM of dNTPs and 0.5 units of Taq polymerase (Tiangen Company, Beijing, China). The reaction mixture was incubated at 94 °C for an initial 4 min, followed by 30 cycles of 94 °C 30 s, 55 °C 30 s and 72 °C 30 s, and a final extension step of 5 min at 72 °C. PCR products were separated on 8% non-denaturing polyacrylamide gel and detected using the silver staining method.

### 4.5. Linkage Analysis

A linkage map was constructed on the basis of genetic linkage between the genotype of SSR markers and seed shattering phenotype in the BC_4_F_2_ population.

### 4.6. Sequencing

In order to compare the sequence difference of *SH4* between Yundao 1 and NIL-*hs1*, the primer (SH4-F: CCGAACACCAAACGCCTCAG, SH4-R: CCGTACTCCCAATACTCGCAGA) was designed on the 5′UTR and 3′UTR region of *SH4* gene for amplifying the target sequence. PCR mixture (25 uL) contained 0.4 mM of each dNTP, 0.3 uM of each primer, 0.5 units of Taq polymerase (KOD FX DNA polymerase, Toyobo, Japan) and template DNA 100 ng in the GeneAMP PCR system 9700 (Applied Biosystems, Foster City, CA, USA). The PCR program was 94 °C for 2 min, followed by 30 cycles at 98 °C for 10 s, 55 °C for 30 s and 68 °C for 2 min. PCR products were separated in 1% agarose gels.

### 4.7. Haplotype Analysis of the SH4 Gene

To analyze the haplotype of the *SH4* gene, the nucleotide sequence of *SH4* in the AA genome was downloaded from the GenBank database, wild rice genome project and public data [5,7,15,23,24,25], 2 SNPs of G237T and C760T were analyzed in 279 rice accessions.

## Figures and Tables

**Table 1:** The haplotypes of the *SH4* at G237T and C760T sites in the AA genome species of genus *Oryza.*

Species	G237T	C760T	No. of Accessions (Varieties)	Phenotype
*O. longistaminata*	G	C	2	Shattering
*O. barthii*	G	C	22	Shattering
	G	T	6	Non-shattering
*O. glaberrima*	G	C	8	Shattering
	G	T	111	Non-shattering
*O. glumaepatula*	G	C	2	Shattering
*O. meridionalis*	G	C	2	Shattering
*O. nivara*	G	C	20	Shattering
	T	C	5	Non-shattering
*O. rufipogon*	G	C	30	Shattering
	T	C	6	Non-shattering
*O. sativa*, *temperate japonica*	T	C	30	Non-shattering
*O. sativa*, *tropical japonica*	T	C	5	Non-shattering
*O. sativa*, *indica*	T	C	25	Non-shattering
*O. sativa*, *aus*	T	C	5	Non-shattering

## Supplementary Materials

The following are available online at https://www.mdpi.com/2223-7747/8/11/475/s1, Figure S1. Frequency distributions of seed shattering rate and the sequence alignment of *HS1*. Figure S2. The information on haplotypes in *SH4* of 279 accessions in AA genome *Oryza*. Table S1. The seed shattering rate of Yundao 1, NIL-*hs1* and F1 hybrid.

## Abbreviations

NIL: Near isogenic line; AL: Abscission layer, V: Vascular bundle.
