# Supplementary material for: Two SNP Mutations Turned off Seed Shattering in Rice
Source: Plants (Basel). 2019 Nov 6;8(11):475. doi: 10.3390/plants8110475 (PMC6918406; doi:10.3390/plants8110475)
Supplement: Supplementary file 1 [file plants-08-00475-s001.zip › Supplementary file S1. Frequency distributions of seed shattering rate and the sequence alignment of SH4.docx]

**Figure S1.** The frequency distributions of seed shattering rate in BC_4_F_2_ population.

**Table S1.** The seed shattering rate of Yundao 1, NIL-*hs1* and F_1_ hybrid.

| **Lines** | **Seed Shattering Rate（%）** | ***P*** |
| --- | --- | --- |
| Yundao1 | 3.66 ± 1.89 | 1.04E-13 |
| NIL-*hs1* | 2.92 ± 1.66 |  |
| F_1_ | 72.34 ± 17.28 |  |


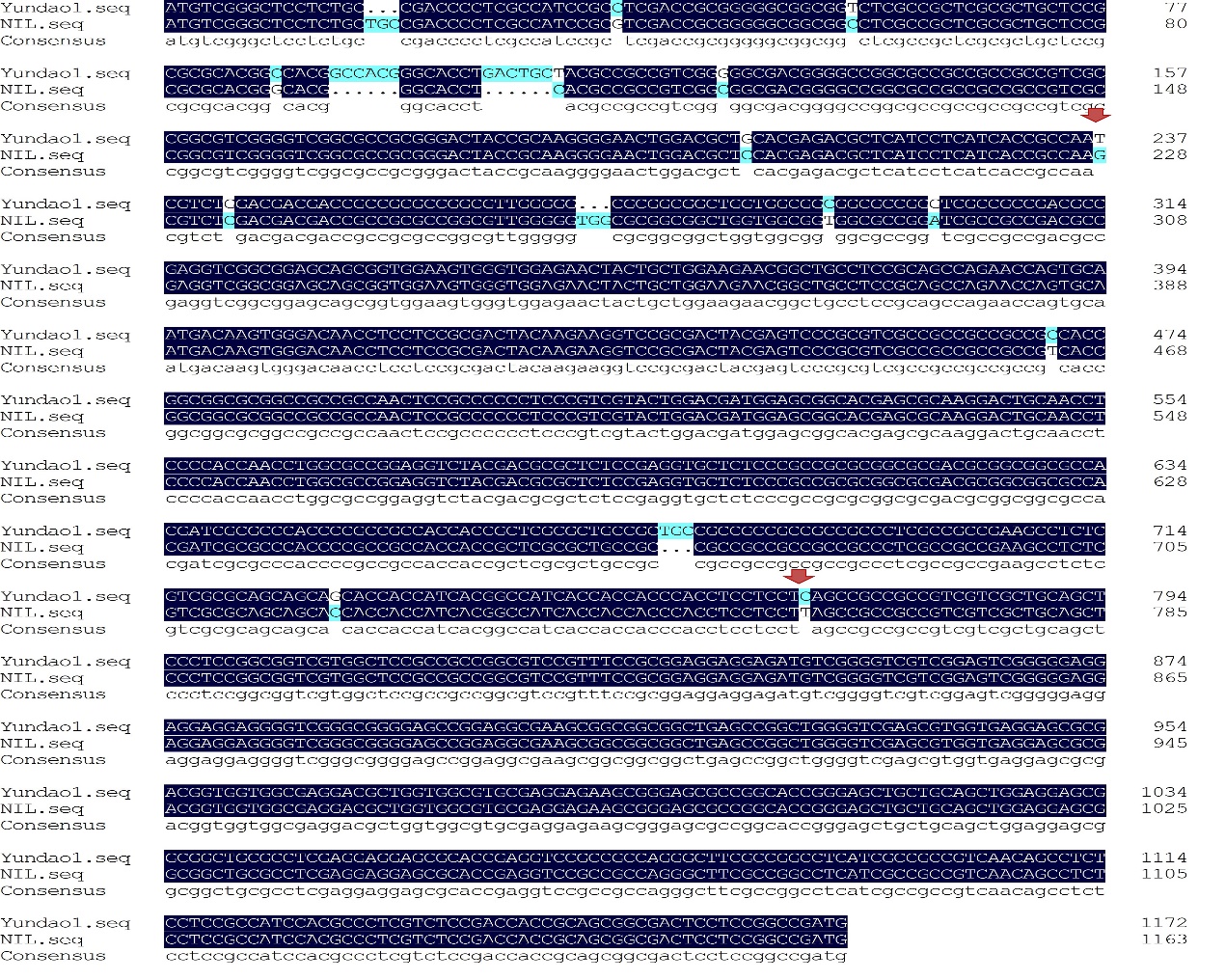


**Figure S2.** Genome sequence alignment of *HS1*. The arrow indicated the site of G237T and C760T.


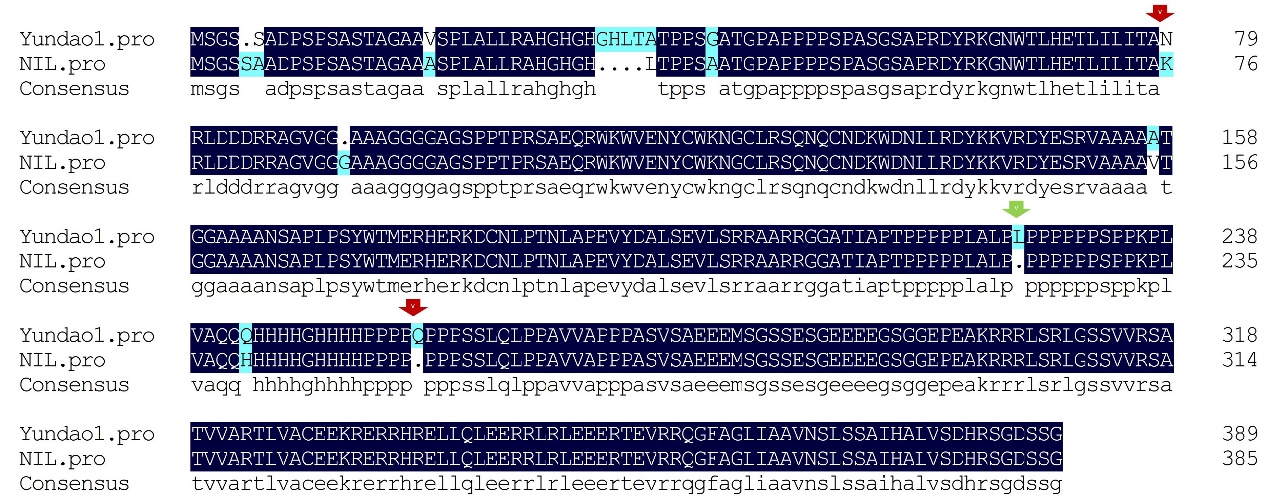


**Figure S3.** Amino acid sequence alignment of *HS1*. The red arrow indicated the critical mutation site in NIL-*hs1*, the green arrow showed the unique amino acid deletion in NIL, compared with amino acid sequence of *HS1* in AA genome species.
